# Asphyxia at birth affects brain structure in patients on the schizophrenia-bipolar disorder spectrum and healthy participants

**DOI:** 10.1017/S0033291720002779

**Published:** 2022-04

**Authors:** Laura Anne Wortinger, Kristine Engen, Claudia Barth, Ole A. Andreassen, Kjetil Nordbø Jørgensen, Ingrid Agartz

**Affiliations:** 1 Department of Psychiatric Research, Diakonhjemmet Hospital, Oslo, Norway; 2 NORMENT, Institute of Clinical Medicine, University of Oslo, Oslo, Norway; 3 Division of Mental Health and Addiction, NORMENT, Oslo University Hospital, Oslo, Norway; 4 Department of Clinical Neuroscience, Centre for Psychiatric Research, Karolinska Institute, Stockholm, Sweden

**Keywords:** Asphyxia, bipolar disorder, caudate, intracranial volume, schizophrenia, surface area

## Abstract

**Background:**

Uncertainty exists about what causes brain structure alterations associated with schizophrenia (SZ) and bipolar disorder (BD). Whether a history of asphyxia-related obstetric complication (ASP) – a common but harmful condition for neural tissue – contributes to variations in adult brain structure is unclear. We investigated ASP and its relationship to intracranial (ICV), global brain volumes and regional cortical and subcortical structures.

**Methods:**

A total of 311 patients on the SZ – BD spectrum and 218 healthy control (HC) participants underwent structural magnetic resonance imaging. They were evaluated for ASP using prospective information obtained from the Medical Birth Registry of Norway.

**Results:**

In all groups, ASP was related to smaller ICV, total brain, white and gray matter volumes and total surface area, but not to cortical thickness. Smaller cortical surface areas were found across frontal, parietal, occipital, temporal and insular regions. Smaller hippocampal, amygdala, thalamus, caudate and putamen volumes were reported for all ASP subgroups. ASP effects did not survive ICV correction, except in the caudate, which remained significantly smaller in both patient ASP subgroups, but not in the HC.

**Conclusions:**

Since ASP was associated with smaller brain volumes in all groups, the genetic risk of developing a severe mental illness, alone, cannot easily explain the smaller ICV. Only the smaller caudate volumes of ASP patients specifically suggest that injury from ASP can be related to disease development. Our findings give support for the ICV as a marker of aberrant neurodevelopment and ASP in the etiology of brain development in BD and SZ.

## Introduction

Schizophrenia (SZ) and bipolar disorders (BD) are severe mental disorders with overlapping clinical characteristics and often regarded as part of a clinical continuum (Vieta et al., 2018). They share complex pathophysiology, involving genetic and environmental factors. A key pathophysiological hypothesis for SZ and BD is abnormal neurodevelopment, which seems to fit with several observations of genetic susceptibility (Smeland et al., 2018) and early life stressors such as birth complications, infections, migration, neglect and trauma (Radua et al., 2018).

Accounts of obstetric complications (OCs) often describe the pre- and perinatal environment and comprise a broad range of adversities that can occur during the fetal, perinatal and the neonatal period (McNeil, Cantor-Graae, & Sjostrom, 1994; McNeil & Sjöström, 1995). Although frequent in both healthy and psychiatric populations (Nicodemus et al., 2008; Ursini et al., 2018; Wortinger et al., 2019), OCs have been found to increase the risk of SZ with an odds ratio of 1.5–5 (Cannon, Jones, & Murray, 2002a; Geddes & Lawrie, 1995; Nosarti et al., 2012; Pugliese et al., 2019), but the relationship between OCs and BD is unclear (Nosarti et al., 2012; Pugliese et al., 2019; Scott, McNeill, Cavanagh, Cannon, & Murray, 2006). Genetic risk for developing SZ multiplies by a factor of 5 in the context of severe OCs (Ursini et al., 2018), which suggest a connection between a genetic liability for SZ and vulnerability for adverse events during pregnancy and birth.

Asphyxia is a condition defined as a deficient supply of oxygen to the body. Within labor and delivery complications, perinatal asphyxia is a severe complication with the potential to cause great harm in the offspring, such as neonatal mortality or morbidity with long-term consequences (McNeil & Sjöström, 1995). In a previous study, a history of more than one co-occurring OC was found to be associated with lower cognitive abilities among adult patients with SZ and BD, as well as in healthy controls (Wortinger et al., 2019). Interestingly, asphyxia was prevalent across groups and very common among participants who had experienced several OCs, with a prevalence of 81%. This raised the possibility that neural insult related to asphyxia is consequential for adult brain function and, possibly, structure.

During early childhood, total brain volume (TBV) and intracranial volume (ICV) increase in parallel until early adolescence, wherein TBV appears to be the driving factor for ICV growth (Kiesler & Ricer, 2003). From early adolescence, the development of ICV and TBV diverge; ICV appears to remain static, but TBV decreases during adolescence into adulthood (Tamnes et al., 2017). Childhood head circumference measures are strongly correlated with magnetic resonance imaging (MRI) derived ICV estimates (Hshieh et al., 2016), and ICV might be used as a proxy of early brain development. One longitudinal study found that the rate of cranial growth, measured as head circumference from birth to age 4, predicted later cognitive abilities (Jaekel, Sorg, Baeuml, Bartmann, & Wolke, 2019). These findings substantiate the idea that ICV can inform on the development of brain structure and function.

The discrepancy between ICV and TBV may refer to the normal process of age-related brain maturation and atrophy (Storsve et al., 2014), as well as pathological processes beginning later in life. Several studies have assessed ICV and TBV in relation to cognitive phenotypes in patients with SZ, BD and healthy control participants (Czepielewski, Wang, Gama, & Barch, 2017; Van Rheenen et al., 2018; Woodward & Heckers, 2015). These studies subdivided patients into groups based on current cognitive abilities and an estimate of cognitive abilities preceding the occurrence of disease symptoms. The abnormal cognitive profiles were defined as the neurodevelopmental phenotype, which had low premorbid and current cognitive abilities and exhibited smaller ICV and smaller absolute TBV – indicative of early cerebral disruption or hypoplasia (Czepielewski et al., 2017; Woodward & Heckers, 2015), and the neuroprogressive phenotype, which had only low current cognitive abilities and showed only proportionally smaller TBV in relation to ICV – indicative of excessive brain tissue loss and progressive atrophy (Czepielewski et al., 2017; Van Rheenen et al., 2018; Woodward & Heckers, 2015). The rationale behind these measures is that in the neurodevelopmental phenotype low cognitive abilities and small ICV are consistently present due to halted brain growth, which suggests abnormalities in brain growth and function. Whereas, the neuroprogressive group had only low current cognitive abilities and an ICV that was not different from healthy controls, indicating normal brain growth. Cognitive impairment occurred later and was accompanied by progressive brain tissue loss that is only apparent when adjusting for ICV.

Longitudinal studies (Ducharme et al., 2015; Mills, Lalonde, Clasen, Giedd, & Blakemore, 2014; Raznahan et al., 2011; Tamnes et al., 2017; Wierenga, Langen, Oranje, & Durston, 2014) have shown that cortical surface area (SA) increases during childhood followed by subtle decreases during adolescence, which supports the idea that SA is established early in human development. Whereas, cortical thickness (CT) decreases during adolescence and appears to be the main contributor to TBV reductions (Storsve et al., 2014; Tamnes et al., 2017). Fetal hypoxia, caused by an inadequate supply of oxygen to the fetus due to several reasons during pregnancy and labor (e.g. birth or neonatal asphyxia, umbilical cord abnormalities, placental infarcts, third-trimester bleeding, preeclampsia, etc.), was associated with smaller SA, but not with CT in a first-episode psychosis study (Smith et al., 2015). The relationship between fetal hypoxia and regional SA and CT has not been previously reported. Putative associations between fetal hypoxia and subcortical structures have shown mixed results with smaller hippocampal and amygdala volumes reported in SZ and BD (Haukvik et al., 2014a; Van Erp et al., 2002) and larger hippocampal volumes reported in SZ (Haukvik et al., 2010). A study comparing SZ patients with their siblings and unrelated healthy controls showed a significant relationship between fetal hypoxia and smaller gray matter volume (but not white matter volume) in both patients and their relatives, which was not found in the healthy controls (Cannon et al., 2002b). Extensive studies on hypoxic-ischemic injury have reported neuronal death, reduced neural growth processes and white matter damage in infants (Rees & Inder, 2005; Volpe, 2012), but altered white matter volume associated with fetal hypoxia has not been identified in SZ.

Using prospective birth registry data, we were able to show a connection between OCs and a neurodevelopmental phenotype characterized by lower premorbid and current cognitive abilities (Wortinger et al., 2019). Here we investigated whether the relationship extends to neuroimaging measures of brain structure. Specifically, we explored whether a history of asphyxia-related OCs (ASP) is associated with adult brain measures in patients with severe mental illness and healthy controls. The aims of this study were to: (1) investigate the prevalence of ASP across diagnoses; (2) examine the effect of a history of ASP on ICV, TBV, global brain structural volumes, total cortical surface area and mean cortical thickness and regional brain measures in ASP defined subgroups; and (3) determine if effects of ASP are more pronounced in SZ or BD.

## Methods and Materials

### Participants

The Thematically Organized Psychosis (TOP) study is a thematic research effort focused on the disease mechanisms of psychotic disorders and is the main study protocol at the Norwegian Centre for Mental Disorders Research (NORMENT, Oslo, Norway; www.med.uio.no/norment/english). Adult patients with schizophrenia or bipolar spectrum disorders were recruited consecutively from out- and inpatient psychiatric units of public hospitals in the Oslo region. The hospitals are located in different parts of the city and are representative of the city's variation in sociodemographic characteristics. The healthy controls (HC) were randomly selected from the national population register and were residents in the same catchment area as the patients. After a complete description of the study, all participants gave written informed consent. The Regional Committee for Research Ethics and the Norwegian Data Inspectorate approved the study. Participant inclusion for this study was carried out between 2002 and 2012 in accordance with the Declaration of Helsinki.

Exclusion criteria for both patients and HC were hospitalization for previous moderate or severe head injury, neurological disorder, medical conditions thought to interfere with brain function and age outside the range of 18–65 years. Additional exclusion criteria for HC were current or previous somatic illness and substance misuse disorders or dependency within the last 6 months. HC were also excluded if they or a first-degree relative had a lifetime history of severe psychiatric disorder.

All patients underwent a thorough clinical investigation by trained psychologists and physicians. Clinical diagnoses were assessed using the Structured Clinical Interview for DSM-IV axis 1 disorder (SCID-I) module A-E (Spitzer, Williams, Gibbon, & First, 1988). Psychosocial function was assessed with the Global Assessment of Function scale, split version [GAF; (Pedersen, Hagtvet, & Karterud, 2007)]. Current psychotic symptoms were rated by the use of the Positive and Negative Syndrome Scale [PANSS; (Kay, Fiszbein, & Opler, 1987)].

HC were interviewed by trained research assistants and examined with the Primary Care Evaluation of Mental Disorders (Prime-MD) to ensure no current or previous psychiatric disorders (Spitzer et al., 1994).

For the current study, participants were included if they had both OC and MRI data. The total subject sample (*n* = 529) consisted of patients with a DSM-IV diagnosis within the *schizophrenia spectrum* (SZ): schizophrenia (DSM-IV 295.1, 295.3, 295.6 and 295.9; *n* = 106), schizophreniform disorder (DSM-IV 295.4; *n* = 16), schizoaffective disorder (DSM-IV 295.7; *n* = 14) or psychosis not otherwise specified (DSM-IV 298.9; *n* = 60); or within the *bipolar disorder spectrum* (BD): Bipolar I disorder (DSM-IV 296.0–7; *n* = 72), Bipolar II disorder (DSM-IV 296.89; *n* = 36) or bipolar disorder not otherwise specified (DSM-IV 296.80; *n* = 7); and HC (*n* = 218).

### Obstetric complications

Birth data were collected from the Medical Birth Registry of Norway (MBRN). In Norway, there is mandatory reporting on all births after gestational week 16. MBRN data were scored for the presence and severity of OCs (McNeil & Sjöström, 1995) by KE.

The validated McNeil–Sjöström scale (McNeil et al., 1994; McNeil & Sjöström, 1995) includes several hundred events of potential harm to the fetus/offspring, each classified according to the severity on an ordinal scale from 1 to 6. As in other reports (Nicodemus et al., 2008; Ursini et al., 2018; Wortinger et al., 2019), an incidence of severe OCs was reported in those participants who had experienced one or more complications of a grade 5 or 6. Complications below grade 5 have the potential to cause harm, but to a lesser extent (McNeil et al., 1994). Examples of grade 3 complications are conditions like non-preeclamptic hypertension, polyhydramnios and hyperemesis and examples of grade 4 complication are conditions like mild preeclampsia, oligohydramnios and breech delivery (McNeil et al., 1994). We classified participants with complications of grade 4 and below as having an absence of severe OCs. A complication of grade 5 is defined as an event that is ‘potentially clearly greatly relevant/harmful’ to the central nervous system of the developing fetus/offspring and a complication of grade 6 is defined as an event that causes ‘very great harm to or deviation in offspring’ (McNeil & Sjöström, 1995). Examples of grade 5 or 6 are as follows: severe preeclampsia, bleeding before 28 weeks, asphyxia, discolored placenta/amniotic fluid, emergency caesarean delivery, preterm birth ⩽35 weeks, low Apgar score (0–3 at 1 min or 0–7 at 5 min), bleeding during labor, low birth weight ⩽2000 g and eclampsia.

The ASP variable was defined as having had a report of a complication coded AS53 (asphyxia without other signs), AS54 (asphyxia with poor fetal sound), AS55 (asphyxia and discolored amniotic fluid), AS56 (asphyxia with poor fetal sound and discolored amniotic fluid) or AS61 (asphyxia) on the MBRN registry form, all of which state the presence of ASP at birth. See Wortinger et al. (2019) for MBRN form. OC co-occurrence was defined as having experienced more than one severe OC during pregnancy and birth.

### Image acquisition and processing

All participants underwent MRI scanning on a 1.5 T Siemens Magnetom Sonata scanner (Siemens Medical Solutions, Erlangen, Germany) equipped with an 8-channel head coil. After a conventional 3-plane localizer, two sagittal T1-weighted magnetization prepared rapid gradient echo (MPRAGE) volumes were acquired with the Siemens tfl3d1_ns pulse sequence (TE = 3.93 ms, TR = 2730 ms, TI = 1000 ms, flip angle = 7°; FOV = 24 cm, voxel size = 1.33 × 0.94 × 1 mm^3^, number of partitions = 160).

The Freesurfer software package (version 5.3) was used to process images with volume and surface-based streams (Dale, Fischl, & Sereno, 1999; Fischl et al., 2002). Post-processed images received a quality control of surface reconstruction and subcortical segmentations, and if deemed necessary, manual editing was performed by trained assistants following standard FreeSurfer procedures (McCarthy et al., 2015). Volume-based analyses were used to extract mean volume estimates for intracranial volume (ICV; estimate based on the Talairach transform), total brain volume (TBV; brain segmentation volume, without ventricles), white and gray matter volumes, subcortical volumes and cortical surface area across the whole brain. Surface-based analyses were used to extract cortical thickness measurements by reconstructing a 3-dimensional cortical surface model.

### Statistical analysis

Statistical analyses were performed within the Statistical Package of Social Sciences (SPSS), version 25 (IBM, USA; www.spss.com). Demographic and clinical characteristics were compared between groups using χ^2^ tests for categorical variables and analysis of variance (ANOVA) or covariance (ANCOVA) for continuous variables. Subjects with missing data on any particular variable were omitted from analyses involving that variable but were included in analyses for which all required variables were present.

To evaluate the contribution of OCs other than ASP on global brain measures, we assessed ICV, TBV, global brain structural volumes, total cortical surface area and mean cortical thickness in a full factorial 3 × 3 ANCOVA with OCs [OCs with ASP (OC, ASP+); OCs other than ASP (OC, ASP−); no OCs present (no OC)] and the group included as between-group factors, covarying for age and sex. Since OC, ASP− was not associated with global brain measures, the variable OCs, ASP− was not included in further analyses.

We assessed ICV, TBV, white and gray matter volumes, total subcortical volume, total cortical surface area and mean cortical thickness using a full factorial 2 × 3 ANCOVA with ASP (ASP+/ASP−) and group included as between-group factors, covarying for age and sex. A false discovery rate (FDR) of 5% was used to correct for multiple testing of the following comparisons, *separately*: global brain measures – ICV, TBV, total subcortical gray matter volume, total gray matter volume, left, right and whole cortical gray and white matter volumes, total cortical surface area and thickness (12 comparisons); subcortical volumes were assessed for the left and right hippocampus, amygdala, thalamus, caudate, putamen, pallidum, accumbens and cerebellum (16 comparisons); and regional cortical surface area was evaluated on all 34 regions of the left and right hemispheres based on the Desikan-Killiany brain atlas (34 comparisons per hemisphere). In the statistical analysis, ICV was subsequently added to each full factorial model to assess relative differences in those measures showing main effects of ASP. Effect sizes are reported as Cohen's *d* (Nakagawa & Cuthill, 2007).

## Results

### Demographic and clinical variables

Demographic and clinical data are presented in Table 1.

**Table 1.** Demographic and clinical characteristics

	HC	*n* = 218				BD	*n* = 115				SZ	*n* = 196				HC *v.* BD		HC *v.* SZ		BD *v.* SZ	
	ASP−		ASP+		χ^2^*/t*	ASP−		ASP+		χ^2^*/t*	ASP−		ASP+		χ^2^*/t*	χ^2^*/t*	*p*	χ^2^*/t*	*p*	χ^2^*/t*	*p*
	*n* = 186	%	*n* = 32	%		*n* = 93	%	*n* = 22	%		*n* = 175	%	*n* = 21	%							
OC co- occurrence	2	1%	19	59%	106.60***	3	3%	11	50%	36.41***	6	3%	11	52%	56.72***						
Sex (female)	82	44%	12	38%	0.48	54	58%	11	50%	0.47	70	40%	7	33%	0.35	5.42	**0.020**	0.63	0.429	8.68	**0.003**
	Mean	s.d.	Mean	s.d.		Mean	s.d.	Mean	s.d.		Mean	s.d.	Mean	s.d.							
Age (years)	30.68	6.07	28.31	5.79	−2.05	27.77	6.09	27.36	5.84	−0.29	27.45	6.03	26.71	6.51	−0.53	−3.78	**<0.001**	−4.97	**<0.001**	−0.46	0.650
Education (years)	14.41	2.39	14.03	1.98	−0.86	13.18	2.20	14.18	2.06	1.92	12.77	2.51	12.26	2.02	−0.85	−3.53	**<0.001**	−6.93	**<0.001**	−2.33	**0.021**
Birth weight (grams)^¥^ ^a^	3556	507	3428	507	−1.32	3624	545	3477	546	−1.13	3506	544	3137	544	−2.93**	1.32	0.187	−1.48	0.141	−2.22	**0.027**
Gestation age (weeks) ^b^	40	1.75	41	2.48	2.33	40	1.98	41	2.27	1.34	40	1.91	39	4.68	−0.99	0.42	0.672	−0.67	0.501	−0.93	0.356
Birth head circumference (cm)^¥^ ^c^	35.23	1.42	34.96	1.43	−0.79	35.50	1.47	35.12	1.47	−0.85	35.31	1.40	35.11	1.41	−0.44	1.19	0.233	0.89	0.377	−0.29	0.771
Birth length (cm)^¥^ ^d^	50.53	2.13	50.45	2.13	−0.20	50.51	2.93	50.67	2.93	0.23	50.32	2.20	49.46	2.19	−1.58	0.54	0.591	−1.36	0.175	−1.26	0.207
Adult ICV (mm^3^ /1000)^†^ ^e^	1638	136	1608	134	−1.21	1647	135	1581	134	−2.30*	1621	135	1570	134	−1.53	−0.01	0.993	−1.15	0.251	−1.13	0.261
Adult height (cm)^¥^ ^f^	176.85	5.99	173.63	6.01	−2.76**	174.03	6.09	175.74	6.09	1.18	175.35	6.48	172.01	6.48	−2.19*	−0.25	0.805	−3.20	**0.001**	−2.14	**0.033**
Age at illness onset						19.04	6.09	20.23	6.02	0.82	22.33	5.10	22.15	5.82	−0.15					4.64	**<0.001**
PANSS						47.25	10.90	44.36	8.24	1.16	60.31	17.12	57.81	11.50	0.65					−7.69	**<0.001**
General						26.33	6.31	24.18	3.42	1.61	31.12	8.62	30.90	6.71	0.11					−5.66	**<0.001**
Negative						10.65	3.70	10.27	3.68	0.43	14.91	6.58	13.24	4.70	1.10					−6.26	**<0.001**
Positive						10.27	3.91	9.91	3.77	0.48	14.29	5.13	13.67	4.63	0.58					−7.25	**<0.001**
GAF-S						55.20	11.43	60.32	12.21	−1.86	43.39	11.76	49.10	11.98	−2.09					8.77	**<0.001**
GAF-F						53.06	12.59	57.86	13.54	−1.59	45.70	11.83	47.90	12.41	−0.80					5.59	**<0.001**
APD dose (CPZ equiv.)						231.6	222.2	215.2	93.2	0.24	364.3	296.5	281.1	173. 5	1.10					−3.13	**0.002**
*n* and % using						*n*	%	*n*	%		*n*	%	*n*	%							
						47	51%	11	50%	0.00	153	87%	16	76%	1.99					47.09	**<0.001**
Lithium						18	19%	3	14%	0.39											
Psychotic symptoms						56	60%	13	59%	0.92											

s.d., standard deviation; HC, healthy controls; BD, bipolar spectrum; SZ, schizophrenia spectrum; PANSS, Positive and Negative Syndrome Scale; GAF-S, Global Assessment of Functioning- symptoms; GAF-F, Global Assessment of Functioning- functioning; APD, antipsychotic drug; CPZ, chlorpromazine. Bold values denote statistical significance at the *p* < 0.05 level.

¥ Means are adjusted for sex.

† Means are adjusted for age and sex.

* *p* < 0.05; ** *p* < 0.01; *** *p* < 0.001.

a Total sample size (HC: ASP− *n* = 186, ASP+ *n* = 32; BD: ASP− *n* = 93, ASP+ *n* = 22; SZ: ASP− *n* = 174, ASP+ *n* = 21).

b Total sample size (HC: ASP− *n* = 175, ASP+ *n* = 30; BD: ASP− *n* = 88, ASP+ *n* = 21; SZ: ASP− *n* = 167, ASP+ *n* = 18).

c Total sample size (HC: ASP− *n* = 89, ASP+ *n* = 22; BD: ASP− *n* = 53, ASP+ *n* = 14; SZ: ASP− *n* = 99, ASP+ *n* = 11).

d Total sample size (HC: ASP− *n* = 184, ASP+ *n* = 32; BD: ASP− *n* = 90, ASP+ *n* = 21; SZ: ASP− *n* = 167, ASP+ *n* = 18).

e Total sample size (HC: ASP− *n* = 186, ASP+ *n* = 32; BD: ASP− *n* = 93, ASP+ *n* = 22; SZ: ASP− *n* = 175, ASP+ *n* = 21).

f Total sample size (HC: ASP− *n* = 183, ASP+ *n* = 31; BD: ASP− *n* = 92, ASP+ *n* = 22; SZ: ASP− *n* = 170, ASP+ *n* = 20).

### Obstetric complications

There were no significant differences in the frequency of OCs between patient groups and HC, but it did differ between the two patient groups (Table 2). ASP was significantly more prevalent in the BD group compared to the SZ group. Low birth weight was more frequent in the SZ group compared to the HC. Bleeding during labor occurred more often in the HC in comparison to the SZ group. The most frequent complications across groups were ASP (⩾44%) and discolored placenta/amniotic fluid (⩾41%). There were significantly more cases of OC co-occurrence in the presence of ASP than when ASP was not present (Table 1). Discolored placenta/amniotic fluid was the most common co-occurring severe OC with ASP, present in 31 out of 41 cases. Low birthweight co-occurred with ASP in three out of 41 cases.

**Table 2.** Frequency and type of obstetric complications using χ^2^ tests

	HC		BD		SZ		HC *v*. BD		HC *v*. SZ		BD *v*. SZ	
	*n* = 218		*n* = 115		*n* = 196		χ^2^	*p*	χ^2^	*p*	χ^2^	*p*
	*n*		*n*		*n*							
***Obstetric complications*** ^a^	61	28%	42	37%	48	24%	2.57	0.109	0.65	0.421	5.10	**0.024**
**Asphyxia**^b^	32	52%	22	52%	21	44%	1.10	0.295	1.45	0.228	4.31	**0.038**
Discolored placenta/amniotic fluid ^b^	25	41%	20	48%	20	42%	2.26	0.133	0.17	0.680	3.34	0.068
**Bleeding during labor** ^b^	12	20%	6	14%	3	6%	0.01	0.912	4.67	**0.031**	3.51	0.061
Preterm ⩽35 weeks ^b^	6	10%	3	7%	7	15%	0.01	0.939	0.23	0.633	0.22	0.642
Other birth complications ^b^	4	7%	3	7%	7	15%	0.22	0.640	1.2	0.273	0.22	0.642
Bleeding before 28 weeks ^b^	3	5%	1	2%	7	15%	0.16	0.687	2.11	0.146	2.11	0.146
Low Apgar score ^b^	2	3%	1	2%	2	4%	0.00	0.965	0.01	0.915	0.02	0.895
Emergency C-section ^b^	1	2%	1	2%	3	6%	0.21	0.645	1.24	0.266	0.25	0.617
**Low birth weight ⩽2000 g** ^b^	0	0%	2	5%	4	8%	3.81	0.051	4.52	**0.034**	0.04	0.847

HC, healthy controls; BD, bipolar spectrum; SZ, schizophrenia spectrum. Bold values denote statistical significance at the *p* < 0.05 level.

a % refers to the number of cases with obstetric complications in relation to the group sample size.

b % refers to the number of cases with the specific complication in relation to the total number of obstetric complications within each group.

### ASP effects on global brain measures

We found that OC, ASP+ was specifically related to ICV and other brain measure differences (see online Supplementary materials Table S1) and OC, ASP− was not related to any of the brain measures. In the full factorial model, there was a significant main effect of OCs on ICV after controlling for the effect of age and sex, *F*_(2,518)_ = 4.32, *p* = 0.014. Pairwise comparisons revealed that adult ICV after having experienced OC, ASP+ was significantly smaller than when no OCs were present (*p* = 0.006) and ICV was smaller with OC, ASP+ than having experienced severe OC, ASP− (*p* *=* 0.011). The presence of an OC, ASP− did not show a significant difference in ICV compared to those with no OCs (*p* = 0.601). The covariate, sex, was significantly related to ICV, *F*_(1,518)_ = 361.24, *p* < 0.001, wherein females had smaller ICV than males. There were no significant OCs by group interactions on any of the brain measures. Since OC, ASP− was not associated with ICV or other global brain measures, the variable OCs, ASP− was not included in further analyses.

**Fig. 1.**
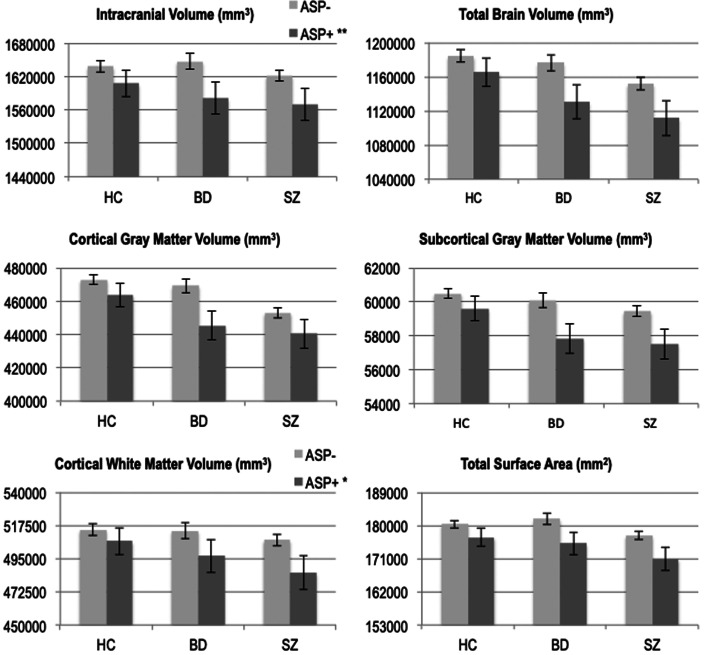
ICV, TBV, global brain structural volumes and total surface area estimate in health controls (HC) and patients within the schizophrenia spectrum (SZ) and bipolar disorder spectrum (BD), *unadjusted* for ICV. *Top panels*: Absolute ICV and TBV corrected means were significantly decreased in all adult participant subgroups that had experienced asphyxia at birth. *Middle panels*: Cortical and subcortical gray matter corrected volumes were significantly decreased in all adult participant subgroups that had experienced asphyxia at birth. *Bottom panel*: Cortical white matter volume and total surface area values were significantly decreased in all adult participant subgroups that had experienced asphyxia at birth. Main effect of asphyxia was significant at *p* < 0.05 for cortical white matter volume, and all other values had a significance of *p* < 0.005. Measures were corrected for age and sex. Error bars = standard error of mean. ** *p* < 0.005, * *p* < 0.05.

There was a significant main effect of ASP that survived the FDR correction rate of 5% for all brain measures, except total cortical thickness (Fig. 1 and online Supplementary materials Table S2). ICV and absolute values of TBV; total subcortical gray matter volume; total gray matter volume; left, right and whole cortical gray and white matter volumes; total cortical surface area was smaller in the three ASP+ subgroups within SZ, BD and HC. There were no significant ASP by group interactions on any of the global brain measures. There was no effect of ASP on any of these global measures after adjusting for ICV.

### Regional cortical surface area estimates

**Fig. 2.**
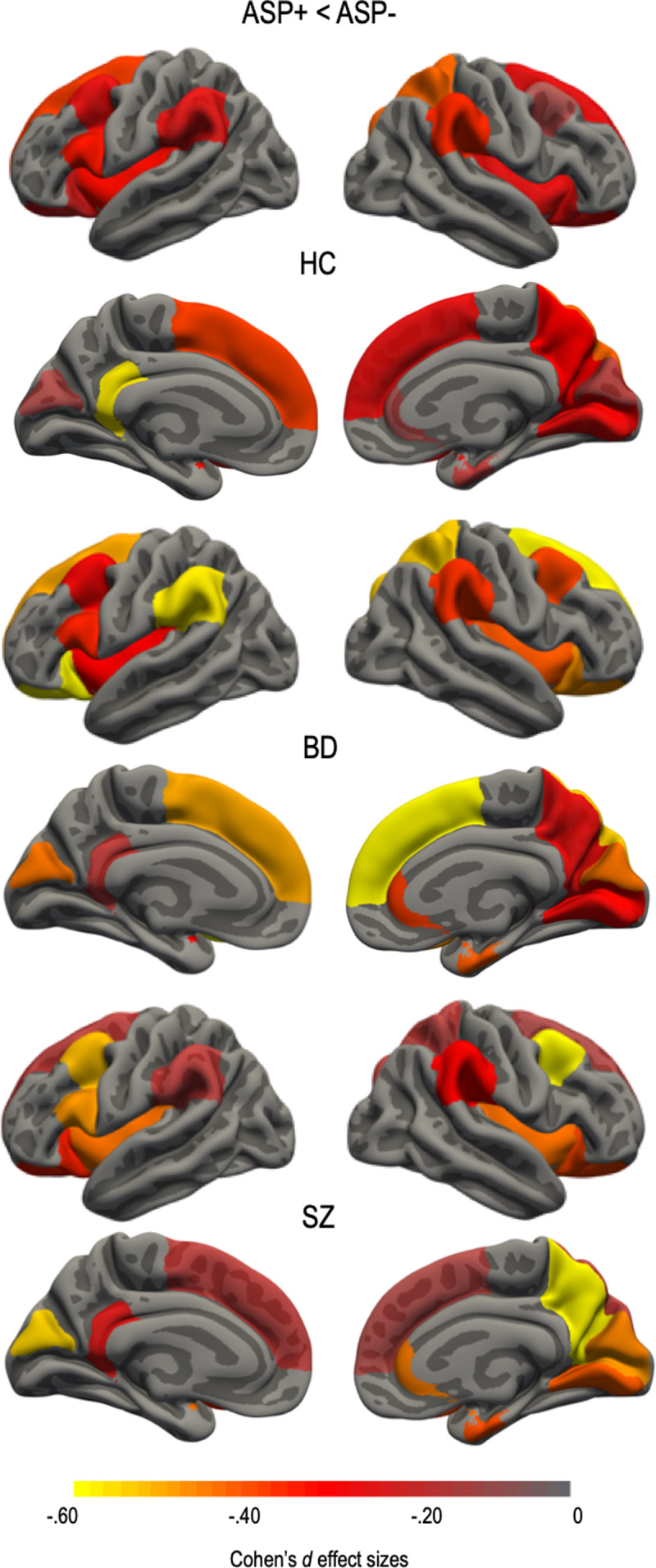
Asphyxia **(**ASP+) effect size differences for regions surviving false discovery rate (FDR) correction of 5% in health controls (HC) and patients within the schizophrenia spectrum (SZ) and bipolar disorder spectrum (BD). Regional cortical surface area was smaller in all ASP+ subgroups. Regions that did not survive correction were assigned a value of 0. Effect sizes were corrected for age and sex. Color bar represents Cohen's *d* values with ASP+ subgroups showing smaller surface area compared to the ASP− subgroups.

There was a significant main effect of ASP that survived the FDR correction rate of 5% for 20 regional surface area measures (Fig. 2 and online Supplementary materials Table S3). Absolute regional values of the left and right superior frontal; left and right caudal middle frontal; left pars opercularis; left and right lateral orbitofrontal; right rostral anterior cingulate; right superior parietal; left and right supramarginal; right precuneus; left isthmus cingulate; right entorhinal; right lingual; left and right cuneus; right pericalcarine; and left and right insulae surface area estimates were smaller in the ASP+ subgroups. There were no significant ASP by group interactions on any of the surface area estimates. There was no significant effect of ASP on any surface area after adjusting for ICV.

### Subcortical volumes

**Fig. 3.**
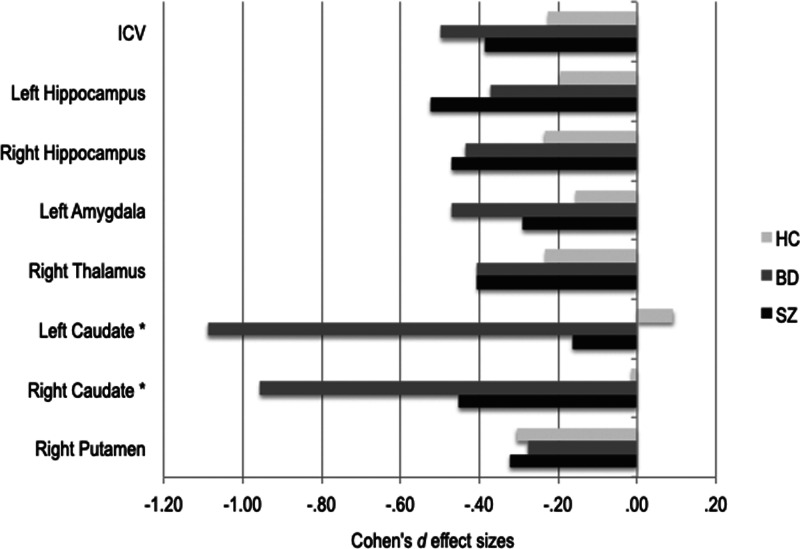
Cohen's *d* effect sizes for ICV and subcortical volume differences between asphyxia-defined subgroups of health controls (HC) and patients within the schizophrenia spectrum (SZ) and bipolar disorder spectrum (BD). Within each patient and HC group, ASP+ *v.* ASP− effect size differences are shown for ICV and subcortical volumes surviving false discovery rate (FDR) correction of 5%. Smaller ASP+ volumes were observed in all groups, except for left and right caudate in the HC. *An interaction effect indicated that within both patient groups, ASP+ subgroups had smaller left and right caudate compared to ASP− subgroups, but this was not the case for the HC group. Effect sizes were corrected for age and sex.

There was a significant main effect of asphyxia that survived the FDR correction rate of 5% for 7 of the 16 subcortical volumes (Fig. 3 and online Supplementary materials Table S4). Absolute values of the left and right hippocampus; left amygdala; right thalamus; left and right caudate; and right putamen volumes were smaller in the ASP+ subgroups compared to the ASP− subgroups, except for the caudate volumes in HC compared to both patient groups. An interaction effect indicated that within both patient groups, patients who experienced ASP at birth had smaller left and right caudate volumes compared to patients who did not experience ASP, which was not the case for the HC group, *F*_(2,521)_ = 7.78, *p* < 0.001 and *F*_(2,521)_ = 4.78, *p* = 0.009, respectively. There were no significant ASP by group interactions on the remaining subcortical volumes. Smaller subcortical volumes seem to be explained by smaller ICV, as almost no subgroup differences remained after correcting for ICV. Volumes of the left and right caudate remained significantly smaller in both patient ASP+ subgroups, even after ICV correction, *F*_(2,520)_ = 8.29, *p* < 0.001 and F_(2,520)_ = 4.82, *p* = 0.008, respectively.

## Discussion

Participants with birth asphyxia showed smaller head size (ICV) as well as smaller volume/surface area across a range of global and regional brain structures. The pattern of differences between ASP+ and ASP− subgroups was similar across patient and HC groups, but effects were larger in the patient groups. Of note, this effect was also found when comparing the ASP+ subgroup with participants who had experienced severe OCs other than ASP. Smaller head size in the ASP+ compared to ASP− subgroups explained all but one of the differences found in global and regional brain measures (as findings did not remain significant after adjustment for ICV). The notable exception was the smaller caudate nuclei, where the findings remained significant among patients, also after adjustment for ICV, which may suggest an ASP by illness vulnerability-interaction.

ICV has been postulated as a biomarker of neurodevelopmental origin, and TBV as an indicator of neurodegenerative processes (Woodward & Heckers, 2015). Reports in psychosis research (Czepielewski et al., 2017; Woodward & Heckers, 2015) found smaller ICV and absolute TBV present in patients with low cognitive abilities on both assessments of current and estimated premorbid functioning, signifying hypoplasia and a neurodevelopmental phenotype. While smaller relative TBV (corrected for ICV) was apparent in patients with only low current cognitive abilities (i.e. not premorbid estimates), implying atrophy and a neuroprogressive phenotype (Czepielewski et al., 2017; Van Rheenen et al., 2018). Our previous work on this sample found lower current and premorbid cognitive abilities in the subgroup of SZ patients with OCs, but having experienced more than one severe OC was related to lower cognitive abilities in all groups, with 81% having had experienced ASP (Wortinger et al., 2019). The results here provide evidence that the relationship between having had experienced ASP extends to smaller ICV and absolute TBV and further supports the neurodevelopmental profile in ASP+ subgroups. We did not observe a neuroprogressive phenotype in the ASP- subgroups, as the TBV results did not survive ICV correction.

Small ICV found in SZ is regarded as an indication of impaired neurodevelopment (Haijma et al., 2013; Hulshoff Pol et al., 2012; Okada et al., 2016; van Erp et al., 2016). We found a lower birth weight in the ASP+ subgroup of SZ patients, which might denote that more vulnerable patients present an aberrant development already before experiencing ASP. Additionally, we report that adult height, but not length at birth, was significantly lower in both ASP+ subgroups of HC and SZ, which could indicate an altered developmental trajectory after experiencing ASP. We cannot rule out the possibility that ASP contributes to the smaller ICV and abnormal development commonly reported in SZ, but since smaller ICV and lower adult height was also found in the ASP+ subgroup of HC, differences cannot be explained by genetic risk for disease development, alone.

ASP is one of the most severe forms of OCs (McNeil & Sjöström, 1995), due to the blatant neural harm in offspring. It is well established that ASP causes the neurological sequelae in cerebral palsy (Volpe, 2012). Recurring themes from MRI studies of term newborns with neonatal encephalopathy presumed to be hypoxic-ischemic are the combinations of abnormalities of basal ganglia/thalamus, cerebral cortex, parasagittal (watershed) cortex, white matter and brainstem (Volpe, 2012). In our study, ASP effects were directly related to smaller adult ICV and global brain volumes but only certain regional cortical and subcortical differences. Deficient blood oxygen supply to cortical vascular zones of the three major cerebral vessels might explain the smaller surface areas of the caudal middle frontal, supramarginal and insular regions and to subcortical end zones of short penetrating blood vessels might explain the smaller caudate, putamen and thalamus volumes reported here, making these regions more susceptible to brain structure abnormalities.

We found smaller TBV, white and gray matter volumes in ASP+ subgroups, which are reported in both BD and SZ with larger effect sizes in SZ (Arnone et al., 2009; de Zwarte et al., 2019; Ellison-Wright & Bullmore, 2010; Haijma et al., 2013; Hibar et al., 2018; Hulshoff Pol et al., 2012; Kempton, Geddes, Ettinger, Williams, & Grasby, 2008; McDonald et al., 2004; van Erp et al., 2018). In a large twin study, there was an association between small TBV and genetic risk of developing SZ, wherein lower white matter volume accounted for 94% of significant portions of the phenotypic correlations, and a significant environmental correlation was found for gray matter (van Haren et al., 2012). We found greater differences in gray matter than white matter volumes between the ASP+ and ASP− subgroups. The added effect of ASP might render these subgroups vulnerable to further neurodevelopmental abnormalities that amplify once illness processes have begun, for example, with the putative negative effects of antipsychotic medication on gray matter (Haijma et al., 2013). ASP+ might be an additive risk to a genetic predisposition that operates by impairing both size development (Smeland et al., 2018) and resilience of the fetal brain (Murray, Bhavsar, Tripoli, & Howes, 2017; Nicodemus et al., 2008).

Our finding of ASP being related to total cortical surface area, as opposed to cortical thickness, is consistent with previous OC and brain structure studies in both psychoses (Haukvik et al., 2014b; Smith et al., 2015) and healthy groups (De Bie et al., 2011; Dubois et al., 2008; Neilson et al., 2019; Walhovd et al., 2012), which together supports cortical surface area measures as the appropriate neuroimaging phenotype related to a neurodevelopmental profile. Our results of regionally smaller surface areas across frontal, parietal, occipital, temporal and insular lobes are also among regions that represent important nodes in large-scale brain networks, which have been associated with cognitive and affective dysfunction in psychiatric and neurological disorders (Menon, 2011). The insulae, key nodes of the salience network (SN), play a crucial role in the dynamic interactions and regulations of two other core networks (i.e. central executive network; default mode network) important in human cognition; specifically, cognitive control over external stimuli and internal mental processes (Menon, 2011). Impaired SN interactions found in SZ might contribute to psychosis (Supekar, Cai, Krishnadas, Palaniyappan, & Menon, 2019).

ASP+ subgroups revealed differences in hippocampal, amygdala, thalamus, caudate and putamen volumes, from seven out of the 16 subcortical comparisons, which are commonly reported different in SZ and BD studies (de Zwarte et al., 2019; Haijma et al., 2013; Hibar et al., 2016; Okada et al., 2016; van Erp et al., 2018). Caudate volumes were smaller in both ASP+ patient subgroups, which was not the case for HC. The caudate is of particular interest in SZ pathology because it is highly innervated by dopamine neurons and mediates a range of cognitive, motor and language functions impaired in the disorder (Zampieri, Bellani, Crespo-Facorro, & Brambilla, 2014). Specifically, hemispheric specialization of the caudate nucleus and cortical regions with connections to the caudate nucleus was reported to be significantly altered in SZ with disrupted hemispheric coordination (Mueller, Wang, Pan, Holt, & Liu, 2015). This finding suggests that the caudate is central in how efficient the brain is in organizing function to specific hemispheres, which starts early in development (Chi, Dooling, & Gilles, 1977; Sun et al., 2005).

We did not observe differences in the number of patients taking lithium, antipsychotic medication or dose equivalents between the ASP patient subgroups. Even though uncertainty exists about what causes brain structure alterations associated with SZ and BD, we cannot rule out an influence of medication; especially, given that previous research has shown that lithium and antipsychotic medications enlarge thalamic (Haijma et al., 2013; Hibar et al., 2016) and basal ganglia volumes (caudate, putamen and pallidum) (Chakos, Lieberman, Alvir, Bilder, & Ashtari, 1995; Di Sero et al., 2019; Haijma et al., 2013). Taken together, our data suggest that smaller subcortical volumes in ASP+ patients are present early in development and persist with later use of medication. This might be consistent with the notion that smaller basal ganglia volumes could be a marker of medication non-response, a possibility which was suggested by some studies (Buchsbaum et al., 2003; Di Sero et al., 2019; Li et al., 2012).

The use of the Medical Birth Registry of Norway is a major strength of this study, which allows for a precise and prospective assessment of OCs in individuals who later develop psychiatric disorders. Our study shows clinical evidence of early life factors and their possible impact on the developing brains of adult participants, including HC, which is currently missing in the field. Including severe OCs as a component of developmental risk may advance our ability to make prognoses in psychiatric disorders, which might complement genetic risk.

Our findings should be considered in the context of limitations to the exclusion criteria of the TOP study, which precluded the recruitment of HC if they had a first-degree relative with a lifetime history of severe psychiatric disorder. If more genetically varied HC had been included in the study, it is possible that even more pronounced differences in brain structure would have been evident, especially ICV. The BD group had half the sample size of the HC and SZ groups, but they had a higher rate of ASP compared to the SZ group. This disproportion of ASP+ BD patients to the ASP+ SZ patients might have contributed to the greater effects found in caudate volumes.

## Conclusions

Our study raises the question of the role of early-life risk factors in the etiology of brain development in BD and SZ. When we examined the differences in brain structure between ASP subgroups of participants, we found that the greatest disparity in measures was between ASP− and ASP+ patient subgroups, and that similar differences were present in HC. This suggests that ASP+ have contributed to smaller brain structures in all groups, which is a finding not easily explained by genetic loading for psychiatric disorders alone. Specifically, our findings give support for the ICV as a marker of aberrant neurodevelopment and the caudate nuclei as a marker for disease development. A better understanding of ASP as a context to brain development could advance the role of perinatal care for reducing the burden of severe mental illness. New strategies for neuroprotective therapies during pregnancy and birth, particularly in women with or genetic risk for mental illness, can be a major form of primary prevention of schizophrenia.

## Supplementary material

For supplementary material accompanying this paper visit https://doi.org/10.1017/S0033291720002779. click here to view supplementary material
