# Supplementary material for: Asphyxia at birth affects brain structure in patients on the schizophrenia-bipolar disorder spectrum and healthy participants
Source: Psychol Med. 2020 Aug 10;52(6):1050–9. doi: 10.1017/S0033291720002779 (PMC9069351; doi:10.1017/S0033291720002779)
Supplement: Supplementary file 1 [file S0033291720002779sup001.docx]

# Asphyxia at birth affects brain structure in patients on the schizophrenia-bipolar disorder spectrum and healthy participants

Laura Anne Wortinger * ^❖^ ^1, 2^, Kristine Engen ^❖^ ^1, 2^, Claudia Barth ^2^, Ole A. Andreassen ^2,3^, Kjetil Nordbø Jørgensen ^1, 2^ and Ingrid Agartz ^1, 2, 4^

^1^Department of Psychiatric Research, Diakonhjemmet Hospital, Oslo, Norway

^2^NORMENT, Institute of Clinical Medicine, University of Oslo, Oslo, Norway

^3^NORMENT, Division of Mental Health and Addiction, Oslo University Hospital

^4^Centre for Psychiatric Research, Department of Clinical Neuroscience, Karolinska Institute, Stockholm, Sweden

*To whom correspondence should be addressed; Dr. Laura Anne Wortinger, Department of Psychiatric Research, Diakonhjemmet Hospital, Postbox 23 Vinderen, 0319, Oslo, Norway; phone: +4792282220; e-mail: [l.a.w.bakke@medisin.uio.no](mailto:l.a.w.bakke@medisin.uio.no)

## Supplementary materials:

Table 1: Pairwise comparisons of OC, ASP+; OC, ASP- and no OCs on measures of ICV, TBV, global brain structural volumes and total cortical surface area and thickness estimates

| **Measure** | **ANCOVA^a^** | **Severe OCs** | **Mean difference** | **SE** | **p value** |
| --- | --- | --- | --- | --- | --- |
| ICV | F(2, 518) = 4.32, *p* = .014 | OC, ASP+ vs. No OCs | -47892.81 | 17398.11 | .006 |
|  |  | OC, ASP+ vs. OC, ASP- | -56881.37 | 22148.81 | .011 |
|  |  | OC, ASP- vs. No OCs | 8988.56 | 17182.12 | .601 |
|  |  |  |  |  |  |
| TBV | F(2, 518) = 4.53, *p* = .011 | OC, ASP+ vs. No OCs | -33983.02 | 12125.84 | .005 |
|  |  | OC, ASP+ vs. OC, ASP- | -40922.68 | 15436.90 | .008 |
|  |  | OC, ASP- vs. No OCs | 6939.66 | 11975.30 | .563 |
|  |  |  |  |  |  |
| LH Cortical Gray Matter Volume | F(2, 518) = 5.37, *p* = .005 | OC, ASP+ vs. No OCs | -6847.55 | 2637.36 | .010 |
|  |  | OC, ASP+ vs. OC, ASP- | -10759.11 | 3357.51 | .001 |
|  |  | OC, ASP- vs. No OCs | 3911.56 | 2604.61 | .134 |
|  |  |  |  |  |  |
| RH Cortical Gray Matter Volume | F(2, 518) = 5.71, *p* = .004 | OC, ASP+ vs. No OCs | -7169.91 | 2630.07 | .007 |
|  |  | OC, ASP+ vs. OC, ASP- | -11001.86 | 3348.23 | .001 |
|  |  | OC, ASP- vs. No OCs | 3831.95 | 2597.42 | .141 |
|  |  |  |  |  |  |
| Cortical Gray Matter Volume | F(2, 518) = 5.56, *p* = .004 | OC, ASP+ vs. No OCs | -14017.45 | 5255.38 | .008 |
|  |  | OC, ASP+ vs. OC, ASP- | -21760.97 | 6690.40 | .001 |
|  |  | OC, ASP- vs. No OCs | 7743.51 | 5190.14 | .136 |
|  |  |  |  |  |  |
| LH Cortical White Matter Volume | F(2, 518) = 2.93, *p* = .054 | OC, ASP+ vs. No OCs | -8049.39 | 3379.72 | .018 |
|  |  | OC, ASP+ vs. OC, ASP- | -8051.92 | 4302.59 | .062 |
|  |  | OC, ASP- vs. No OCs | 2.53 | 3337.77 | .999 |
|  |  |  |  |  |  |
| RH Cortical White Matter Volume | F(2, 518) = 2.71, *p* = .067 | OC, ASP+ vs. No OCs | -7749.68 | 3360.98 | .022 |
|  |  | OC, ASP+ vs. OC, ASP- | -7427.56 | 4278.73 | .083 |
|  |  | OC, ASP- vs. No OCs | -322.115 | 3319.26 | .923 |
|  |  |  |  |  |  |
| Cortical White Matter Volume | F(2, 518) = 2.83, *p* = .060 | OC, ASP+ vs. No OCs | -15799.07 | 6731.45 | .019 |
|  |  | OC, ASP+ vs. OC, ASP- | -15479.48 | 8569.54 | .071 |
|  |  | OC, ASP- vs. No OCs | -319.585 | 6647.89 | .962 |
|  |  |  |  |  |  |
| Subcortical Gray Matter Volume | F(2, 518) = 5.19, *p* = .006 | OC, ASP+ vs. No OCs | -1704.64 | 534.63 | .002 |
|  |  | OC, ASP+ vs. OC, ASP- | -1637.38 | 680.61 | .016 |
|  |  | OC, ASP- vs. No OCs | -67.25 | 527.99 | .899 |
|  |  |  |  |  |  |
| Total Gray Matter Volume | F(2, 518) = 5.29, *p* = .005 | OC, ASP+ vs. No OCs | -17331.21 | 6245.27 | .006 |
|  |  | OC, ASP+ vs. OC, ASP- | -24535.87 | 7950.59 | .002 |
|  |  | OC, ASP- vs. No OCs | 7204.66 | 6167.73 | .243 |
|  |  |  |  |  |  |
| Total Surface Area | F(2, 518) = 4.92, *p* = .008 | OC, ASP+ vs. No OCs | -5395.42 | 1845.71 | .004 |
|  |  | OC, ASP+ vs. OC, ASP- | -6478.37 | 2349.70 | .006 |
|  |  | OC, ASP- vs. No OCs | 1082.95 | 1822.80 | .553 |
|  |  |  |  |  |  |
| Total Thickness | F(2, 518) = 1.08, *p* = .341 | OC, ASP+ vs. No OCs | 0.00 | 0.03 | .877 |
|  |  | OC, ASP+ vs. OC, ASP- | -0.03 | 0.03 | .311 |
|  |  | OC, ASP- vs. No OCs | 0.04 | 0.03 | .143 |

The subgroup of participants with OC, ASP+ displayed smaller brain measures, except cortical thickness. The subgroup OCs, ASP- did not differ from those with no OCs on brain measures. There were no significant OCs by group interactions on any of the brain measures.

^a^ Adjusted for age and sex.

Table 2: Asphyxial-birth subgroup comparisons of ICV, TBV, global brain structural volumes and total surface area estimates for measures surviving FDR correction.

| **Measure** | **ANCOVA^a^** | **Subgroup** | **M** | **SD** | ***d^b^*** | **Comparison^c^** |
| --- | --- | --- | --- | --- | --- | --- |
| ICV | *F* (1, 521) = 8.41, *p* = .004 | HC ASP- | 1638099.05 | 136125.84 |  |  |
|  |  | HC ASP+ | 1607614.30 | 133609.52 | -.23 | < HC ASP- |
|  |  | BD ASP- | 1647311.55 | 134578.47 |  |  |
|  |  | BD ASP+ | 1581010.49 | 133661.53 | -.50 | < BD ASP- |
|  |  | SZ ASP- | 1621456.16 | 134649.93 |  |  |
|  |  | SZ ASP+ | 1569648.41 | 133950.30 | -.39 | < SZ ASP- |
|  |  |  |  |  |  |  |
| TBV | *F* (1, 521) = 8.70, *p* = .003 | HC ASP- | 1184421.61 | 94919.06 |  |  |
|  |  | HC ASP+ | 1165347.58 | 93164.45 | -.20 | < HC ASP- |
|  |  | BD ASP- | 1176442.94 | 93840.09 |  |  |
|  |  | BD ASP+ | 1130431.15 | 93200.72 | -.50 | < BD ASP- |
|  |  | SZ ASP- | 1151840.57 | 93889.92 |  |  |
|  |  | SZ ASP+ | 1111539.65 | 93402.07 | -.43 | < SZ ASP- |
| LH Cortical Gray Matter Volume | *F* (1, 521) = 8.38, *p* = .004 | HC ASP- | 236143.70 | 20679.70 |  |  |
|  |  | HC ASP+ | 231393.50 | 20297.43 | -.23 | < HC ASP- |
|  |  | BD ASP- | 234320.92 | 20444.63 |  |  |
|  |  | BD ASP+ | 222539.70 | 20305.33 | -.58 | < BD ASP- |
|  |  | SZ ASP- | 226129.17 | 20455.48 |  |  |
|  |  | SZ ASP+ | 220126.56 | 20349.20 | -.30 | < SZ ASP- |
| RH Cortical Gray Matter Volume | *F* (1, 521) = 9.13, *p* = .003 | HC ASP- | 236781.55 | 20621.22 |  |  |
|  |  | HC ASP+ | 232276.58 | 20240.03 | -.22 | < HC ASP- |
|  |  | BD ASP- | 234991.25 | 20386.81 |  |  |
|  |  | BD ASP+ | 222691.55 | 20247.91 | -.61 | < BD ASP- |
|  |  | SZ ASP- | 226922.46 | 20397.64 |  |  |
|  |  | SZ ASP+ | 220273.35 | 20291.65 | -.33 | < SZ ASP- |
| Cortical Gray Matter Volume | *F* (1, 521) = 8.80, *p* = .003 | HC ASP- | 472925.25 | 41207.02 |  |  |
|  |  | HC ASP+ | 463670.08 | 40445.29 | -.23 | < HC ASP- |
|  |  | BD ASP- | 469312.17 | 40738.61 |  |  |
|  |  | BD ASP+ | 445231.26 | 40461.04 | -.60 | < BD ASP- |
|  |  | SZ ASP- | 453051.64 | 40760.25 |  |  |
|  |  | SZ ASP+ | 440399.92 | 40548.45 | -.31 | < SZ ASP- |
| LH Cortical White Matter Volume | *F* (1, 521) = 5.87, *p* = .016 | HC ASP- | 256712.62 | 26441.90 |  |  |
|  |  | HC ASP+ | 252480.53 | 25953.12 | -.16 | < HC ASP- |
|  |  | BD ASP- | 256568.35 | 26141.33 |  |  |
|  |  | BD ASP+ | 247674.10 | 25963.22 | -.34 | < BD ASP- |
|  |  | SZ ASP- | 253332.93 | 26155.21 |  |  |
|  |  | SZ ASP+ | 242349.88 | 26019.31 | -.42 | < SZ ASP- |
| RH Cortical White Matter Volume | F (1, 521) = 5.42, *p* = .020 | HC ASP- | 257824.53 | 26296.84 |  |  |
|  |  | HC ASP+ | 254361.10 | 25810.74 | -.13 | < HC ASP- |
|  |  | BD ASP- | 257298.08 | 25997.92 |  |  |
|  |  | BD ASP+ | 249016.14 | 25820.79 | -.32 | < BD ASP- |
|  |  | SZ ASP- | 254372.35 | 26011.73 |  |  |
|  |  | SZ ASP+ | 243077.11 | 25876.57 | -.44 | < SZ ASP- |
| Cortical White Matter Volume | *F* (1, 521) = 5.66, *p* = .018 | HC ASP- | 514537.15 | 52666.36 |  |  |
|  |  | HC ASP+ | 506841.63 | 51692.81 | -.15 | < HC ASP- |
|  |  | BD ASP- | 513866.43 | 52067.69 |  |  |
|  |  | BD ASP+ | 496690.25 | 51712.94 | -.33 | < BD ASP- |
|  |  | SZ ASP- | 507705.27 | 52095.35 |  |  |
|  |  | SZ ASP+ | 485426.99 | 51824.66 | -.43 | < SZ ASP- |
| Subcortical Gray Matter Volume | *F* (1, 521) = 10.39, *p* = .001 | HC ASP- | 60492.02 | 4179.26 |  |  |
|  |  | HC ASP+ | 59588.41 | 4102.01 | -.22 | < HC ASP- |
|  |  | BD ASP- | 60093.14 | 4131.75 |  |  |
|  |  | BD ASP+ | 57841.79 | 4103.60 | -.55 | < BD ASP- |
|  |  | SZ ASP- | 59437.02 | 4133.95 |  |  |
|  |  | SZ ASP+ | 57521.90 | 4112.47 | -.47 | < SZ ASP- |
|  |  |  |  |  |  |  |
| Total Gray Matter Volume | *F* (1, 521) = 9.12, *p* = .003 | HC ASP- | 641143.85 | 48931.93 |  |  |
|  |  | HC ASP+ | 630221.75 | 48027.40 | -.22 | < HC ASP- |
|  |  | BD ASP- | 634502.90 | 48375.71 |  |  |
|  |  | BD ASP+ | 606455.66 | 48046.10 | -.59 | < BD ASP- |
|  |  | SZ ASP- | 616445.78 | 48401.39 |  |  |
|  |  | SZ ASP+ | 599796.83 | 48149.90 | -.35 | < SZ ASP- |
|  |  |  |  |  |  |  |
| Total Surface Area | *F* (1, 521) = 9.32, *p* = .002 | HC ASP- | 180408.61 | 14461.93 |  |  |
|  |  | HC ASP+ | 176822.40 | 14194.60 | -.25 | < HC ASP- |
|  |  | BD ASP- | 181791.96 | 14297.53 |  |  |
|  |  | BD ASP+ | 175131.45 | 14200.12 | -.47 | < BD ASP- |
|  |  | SZ ASP- | 177310.64 | 14305.13 |  |  |
|  |  | SZ ASP+ | 170938.75 | 14230.80 | -.45 | < SZ ASP- |

Subgroup of participants who have experienced asphyxial-birth complications (ASP+); subgroup of participants who have not experienced asphyxial-birth complications (ASP-); HC (healthy controls); BD (bipolar disorder spectrum); SZ (schizophrenia spectrum); LH, left hemisphere; RH, right hemisphere

There were no significant ASP by group interactions on any of the brain measures.

^a^ Adjusted for age and sex; All *p* values significant at a false discovery rate of 5%

^b^ *d* = Cohen’s *d* effect sizes for ASP+ within-group

^c^ < ASP- implies significant reductions relative to not having experienced asphyxia-related OCs.

Table 3: Asphyxial-birth subgroup comparisons of regional surface areas surviving FDR correction.

| **Measure** | **ANCOVA^a^** | **Subgroup** | **M** | **SD** | ***d^b^*** | **Comparison^c^** |
| --- | --- | --- | --- | --- | --- | --- |
| LH superior frontal | F (1, 521) = 7.15, *p* = .008 | HC ASP- | 7609.80 | 796.31 |  |  |
|  |  | HC ASP+ | 7311.73 | 781.59 | -0.38 | < HC ASP- |
|  |  | BD ASP- | 7735.65 | 787.26 |  |  |
|  |  | BD ASP+ | 7342.05 | 781.89 | -0.51 | < BD ASP- |
|  |  | SZ ASP- | 7429.37 | 787.68 |  |  |
|  |  | SZ ASP+ | 7319.98 | 783.58 | -0.14 | < SZ ASP- |
|  |  |  |  |  |  |  |
| RH superior frontal | F (1, 521) = 7.44, *p* = .007 | HC ASP- | 7449.22 | 828.22 |  |  |
|  |  | HC ASP+ | 7243.76 | 812.91 | -0.25 | < HC ASP- |
|  |  | BD ASP- | 7635.69 | 818.80 |  |  |
|  |  | BD ASP+ | 7083.98 | 813.22 | -0.68 | < BD ASP- |
|  |  | SZ ASP- | 7283.74 | 819.23 |  |  |
|  |  | SZ ASP+ | 7190.95 | 814.98 | -0.11 | < SZ ASP- |
|  |  |  |  |  |  |  |
| LH caudal middle frontal | F (1, 521) = 7.63, *p* = .006 | HC ASP- | 2444.88 | 360.87 |  |  |
|  |  | HC ASP+ | 2355.08 | 354.20 | -0.25 | < HC ASP- |
|  |  | BD ASP- | 2458.09 | 356.77 |  |  |
|  |  | BD ASP+ | 2360.92 | 354.34 | -0.28 | < BD ASP- |
|  |  | SZ ASP- | 2401.58 | 356.95 |  |  |
|  |  | SZ ASP+ | 2213.38 | 355.10 | -0.53 | < SZ ASP- |
|  |  |  |  |  |  |  |
| RH caudal middle frontal | F (1, 521) = 8.68, *p* = .003 | HC ASP- | 2327.61 | 387.13 |  |  |
|  |  | HC ASP+ | 2287.72 | 379.97 | -0.10 | < HC ASP- |
|  |  | BD ASP- | 2321.58 | 382.73 |  |  |
|  |  | BD ASP+ | 2176.09 | 380.12 | -0.38 | < BD ASP- |
|  |  | SZ ASP- | 2249.95 | 382.93 |  |  |
|  |  | SZ ASP+ | 2006.05 | 380.94 | -0.64 | < SZ ASP- |
|  |  |  |  |  |  |  |
| LH pars opercularis | F (1, 521) = 9.19, *p* = .003 | HC ASP- | 1813.98 | 265.79 |  |  |
|  |  | HC ASP+ | 1726.86 | 260.88 | -0.33 | < HC ASP- |
|  |  | BD ASP- | 1763.09 | 262.77 |  |  |
|  |  | BD ASP+ | 1673.55 | 260.98 | -0.34 | < BD ASP- |
|  |  | SZ ASP- | 1748.69 | 262.91 |  |  |
|  |  | SZ ASP+ | 1622.06 | 261.55 | -0.48 | < SZ ASP- |
|  |  |  |  |  |  |  |
| LH lateral orbitofrontal | F (1, 521) = 9.71, *p* = .002 | HC ASP- | 2815.21 | 271.82 |  |  |
|  |  | HC ASP+ | 2758.58 | 266.81 | -0.21 | < HC ASP- |
|  |  | BD ASP- | 2856.62 | 268.74 |  |  |
|  |  | BD ASP+ | 2685.90 | 266.91 | -0.64 | < BD ASP- |
|  |  | SZ ASP- | 2769.85 | 268.89 |  |  |
|  |  | SZ ASP+ | 2678.38 | 267.48 | -0.34 | < SZ ASP- |
|  |  |  |  |  |  |  |
| RH lateral orbitofrontal | F (1, 521) = 8.51, *p* = .004 | HC ASP- | 2775.43 | 296.19 |  |  |
|  |  | HC ASP+ | 2701.99 | 290.72 | -0.25 | < HC ASP- |
|  |  | BD ASP- | 2800.70 | 292.83 |  |  |
|  |  | BD ASP+ | 2661.60 | 290.83 | -0.48 | < BD ASP- |
|  |  | SZ ASP- | 2708.92 | 292.99 |  |  |
|  |  | SZ ASP+ | 2596.28 | 291.46 | -0.39 | < SZ ASP- |
|  |  |  |  |  |  |  |
| RH rostral anterior cingulate | F (1, 521) = 5.88, *p* = .016 | HC ASP- | 769.46 | 147.62 |  |  |
|  |  | HC ASP+ | 754.11 | 144.88 | -0.10 | < HC ASP- |
|  |  | BD ASP- | 783.09 | 145.94 |  |  |
|  |  | BD ASP+ | 729.16 | 144.94 | -0.37 | < BD ASP- |
|  |  | SZ ASP- | 780.21 | 146.02 |  |  |
|  |  | SZ ASP+ | 714.77 | 145.25 | -0.45 | < SZ ASP- |
|  |  |  |  |  |  |  |
| RH superior parietal | F (1, 521) = 8.41, p = .004 | HC ASP- | 5809.17 | 596.29 |  |  |
|  |  | HC ASP+ | 5557.39 | 585.26 | -0.43 | < HC ASP- |
|  |  | BD ASP- | 5766.29 | 589.51 |  |  |
|  |  | BD ASP+ | 5446.18 | 585.49 | -0.55 | < BD ASP- |
|  |  | SZ ASP- | 5647.77 | 589.82 |  |  |
|  |  | SZ ASP+ | 5568.77 | 586.75 | -0.13 | < SZ ASP- |
|  |  |  |  |  |  |  |
| LH supramarginal | F (1, 521) = 6.29, *p* = .012 | HC ASP- | 4122.91 | 563.91 |  |  |
|  |  | HC ASP+ | 3992.33 | 553.49 | -0.23 | < HC ASP- |
|  |  | BD ASP- | 4190.86 | 557.50 |  |  |
|  |  | BD ASP+ | 3867.91 | 553.70 | -0.59 | < BD ASP- |
|  |  | SZ ASP- | 4123.10 | 557.79 |  |  |
|  |  | SZ ASP+ | 4044.30 | 554.90 | -0.14 | < SZ ASP- |
|  |  |  |  |  |  |  |
| RH supramarginal | F (1, 521) = 7.10, *p* = .008 | HC ASP- | 3938.44 | 521.28 |  |  |
|  |  | HC ASP+ | 3756.48 | 511.65 | -0.35 | < HC ASP- |
|  |  | BD ASP- | 3978.85 | 515.36 |  |  |
|  |  | BD ASP+ | 3794.84 | 511.85 | -0.36 | < BD ASP- |
|  |  | SZ ASP- | 3897.81 | 515.63 |  |  |
|  |  | SZ ASP+ | 3741.14 | 512.95 | -0.31 | < SZ ASP- |
|  |  |  |  |  |  |  |
| RH precuneus | F (1, 521) = 8.99, *p* = .003 | HC ASP- | 4216.94 | 522.41 |  |  |
|  |  | HC ASP+ | 4062.66 | 512.76 | -0.30 | < HC ASP- |
|  |  | BD ASP- | 4239.29 | 516.48 |  |  |
|  |  | BD ASP+ | 4111.47 | 512.96 | -0.25 | < BD ASP- |
|  |  | SZ ASP- | 4199.07 | 516.75 |  |  |
|  |  | SZ ASP+ | 3891.80 | 514.07 | -0.60 | < SZ ASP- |
|  |  |  |  |  |  |  |
| LH isthmus cingulate | F (1, 521) = 6.48, *p* = .011 | HC ASP- | 1084.45 | 185.85 |  |  |
|  |  | HC ASP+ | 976.85 | 182.41 | -0.58 | < HC ASP- |
|  |  | BD ASP- | 1085.07 | 183.74 |  |  |
|  |  | BD ASP+ | 1056.76 | 182.49 | -0.16 | < BD ASP- |
|  |  | SZ ASP- | 1076.00 | 183.83 |  |  |
|  |  | SZ ASP+ | 1033.85 | 182.88 | -0.23 | < SZ ASP- |
|  |  |  |  |  |  |  |
| RH entorhinal | F (1, 521) = 6.31, *p* = .012 | HC ASP- | 367.77 | 83.48 |  |  |
|  |  | HC ASP+ | 354.86 | 81.93 | -0.16 | < HC ASP- |
|  |  | BD ASP- | 367.20 | 82.53 |  |  |
|  |  | BD ASP+ | 333.57 | 81.97 | -0.41 | < BD ASP- |
|  |  | SZ ASP- | 355.62 | 82.57 |  |  |
|  |  | SZ ASP+ | 323.23 | 82.14 | -0.39 | < SZ ASP- |
|  |  |  |  |  |  |  |
| RH lingual | F (1, 521) = 6.21, *p* = .013 | HC ASP- | 3318.32 | 390.52 |  |  |
|  |  | HC ASP+ | 3215.74 | 383.30 | -0.26 | < HC ASP- |
|  |  | BD ASP- | 3288.67 | 386.07 |  |  |
|  |  | BD ASP+ | 3183.49 | 383.45 | -0.28 | < BD ASP- |
|  |  | SZ ASP- | 3188.77 | 386.28 |  |  |
|  |  | SZ ASP+ | 3030.33 | 384.28 | -0.41 | < SZ ASP- |
|  |  |  |  |  |  |  |
| LH cuneus | F (1, 521) = 8.48, *p* = .004 | HC ASP- | 1589.02 | 201.85 |  |  |
|  |  | HC ASP+ | 1565.88 | 198.11 | -0.12 | < HC ASP- |
|  |  | BD ASP- | 1604.08 | 199.56 |  |  |
|  |  | BD ASP+ | 1516.47 | 198.19 | -0.44 | < BD ASP- |
|  |  | SZ ASP- | 1557.20 | 199.66 |  |  |
|  |  | SZ ASP+ | 1446.77 | 198.62 | -0.56 | < SZ ASP- |
|  |  |  |  |  |  |  |
| RH cuneus | F (1, 521) = 6.18, *p* = .013 | HC ASP- | 1637.85 | 202.60 |  |  |
|  |  | HC ASP+ | 1610.22 | 198.85 | -0.14 | < HC ASP- |
|  |  | BD ASP- | 1672.58 | 200.29 |  |  |
|  |  | BD ASP+ | 1603.07 | 198.93 | -0.35 | < BD ASP- |
|  |  | SZ ASP- | 1612.37 | 200.40 |  |  |
|  |  | SZ ASP+ | 1519.96 | 199.36 | -0.46 | < SZ ASP- |
|  |  |  |  |  |  |  |
| RH pericalcarine | F (1, 521) = 7.84, p = .005 | HC ASP- | 1593.72 | 234.59 |  |  |
|  |  | HC ASP+ | 1528.93 | 230.26 | -0.28 | < HC ASP- |
|  |  | BD ASP- | 1588.20 | 231.93 |  |  |
|  |  | BD ASP+ | 1517.66 | 230.35 | -0.31 | < BD ASP- |
|  |  | SZ ASP- | 1544.14 | 232.05 |  |  |
|  |  | SZ ASP+ | 1432.33 | 230.84 | -0.48 | < SZ ASP- |
|  |  |  |  |  |  |  |
| LH insula | F (1, 521) = 5.87, *p* = .016 | HC ASP- | 2170.06 | 218.96 |  |  |
|  |  | HC ASP+ | 2100.89 | 214.91 | -0.32 | < HC ASP- |
|  |  | BD ASP- | 2194.17 | 216.47 |  |  |
|  |  | BD ASP+ | 2130.16 | 214.99 | -0.30 | < BD ASP- |
|  |  | SZ ASP- | 2145.58 | 216.58 |  |  |
|  |  | SZ ASP+ | 2079.06 | 215.46 | -0.31 | < SZ ASP- |
|  |  |  |  |  |  |  |
| RH insula | F (1, 521) = 8.18, *p* = .004 | HC ASP- | 2238.71 | 260.99 |  |  |
|  |  | HC ASP+ | 2174.58 | 256.17 | -0.25 | < HC ASP- |
|  |  | BD ASP- | 2293.30 | 258.03 |  |  |
|  |  | BD ASP+ | 2188.98 | 256.27 | -0.41 | < BD ASP- |
|  |  | SZ ASP- | 2201.52 | 258.17 |  |  |
|  |  | SZ ASP+ | 2089.10 | 256.83 | -0.44 | < SZ ASP- |

Subgroup of participants who have experienced asphyxial-birth complications (ASP+); subgroup of participants who have not experienced asphyxial-birth complications (ASP-); HC (healthy controls); BD (bipolar disorder spectrum); SZ (schizophrenia spectrum); LH, left hemisphere; RH, right hemisphere

There were no significant ASP by group interactions on any of the brain measures.

^a^ Adjusted for age and sex; All *p* values significant at a false discovery rate of 5%

^b^ *d* = Cohen’s *d* effect sizes for ASP+ within-group

^c^ < ASP- implies significant reductions relative to not having experienced asphyxia-related OCs.

Table 4: Asphyxial-birth subgroup comparisons of regional subcortical volumes surviving FDR correction.

| **Measure** | | **ANCOVA^a^** | **Subgroup** | | **M** | | | **SD** | | ***d^b^*** | **Comparison^c^** |
| --- | --- | --- | --- | --- | --- | --- | --- | --- | --- | --- | --- |
| Left Hippocampus | F (1, 521) = 8.15, *p* = .008 | | | HC ASP- | | 4270.28 | 403.08 | |  | |  |
|  |  | | | HC ASP+ | | 4192.23 | 395.62 | | -0.20 | | < HC ASP- |
|  |  | | | BD ASP- | | 4214.06 | 398.49 | |  | |  |
|  |  | | | BD ASP+ | | 4067.10 | 395.78 | | -0.37 | | < BD ASP- |
|  |  | | | SZ ASP- | | 4054.47 | 398.70 | |  | |  |
|  |  | | | SZ ASP+ | | 3846.41 | 396.63 | | -0.52 | | < SZ ASP- |
| Right Hippocampus | F (1, 521) = 8.88, *p* = .003 | | | HC ASP- | | 4339.73 | 405.04 | |  | |  |
|  |  | | | HC ASP+ | | 4245.29 | 397.55 | | -0.23 | | < HC ASP- |
|  |  | | | BD ASP- | | 4282.15 | 400.43 | |  | |  |
|  |  | | | BD ASP+ | | 4109.28 | 397.71 | | -0.44 | | < BD ASP- |
|  |  | | | SZ ASP- | | 4119.11 | 400.65 | |  | |  |
|  |  | | | SZ ASP+ | | 3932.12 | 398.57 | | -0.47 | | < SZ ASP- |
| Left Amygdala | F (1, 521) = 5.72, *p* = .017 | | | HC ASP- | | 1645.03 | 198.90 | |  | |  |
|  |  | | | HC ASP+ | | 1614.48 | 195.22 | | -0.15 | | < HC ASP- |
|  |  | | | BD ASP- | | 1633.34 | 196.63 | |  | |  |
|  |  | | | BD ASP+ | | 1541.72 | 195.29 | | -0.47 | | < BD ASP- |
|  |  | | | SZ ASP- | | 1588.58 | 196.74 | |  | |  |
|  |  | | | SZ ASP+ | | 1531.79 | 195.71 | | -0.29 | | < SZ ASP- |
| Right Thalamus | F (1, 521) = 7.44, *p* = .007 | | | HC ASP- | | 7342.33 | 667.96 | |  | |  |
|  |  | | | HC ASP+ | | 7188.26 | 655.60 | | -0.23 | | < HC ASP- |
|  |  | | | BD ASP- | | 7220.27 | 660.36 | |  | |  |
|  |  | | | BD ASP+ | | 6955.24 | 655.86 | | -0.41 | | < BD ASP- |
|  |  | | | SZ ASP- | | 7088.16 | 660.71 | |  | |  |
|  |  | | | SZ ASP+ | | 6821.78 | 657.27 | | -0.41 | | < SZ ASP- |
| Left Caudate * | F (1, 521) = 9.11, *p* = .003 | | | HC ASP- | | 3707.57 | 413.40 | |  | |  |
|  |  | | | HC ASP+ | | 3745.38 | 405.77 | | 0.09 | | < HC ASP- |
|  |  | | | BD ASP- | | 3797.57 | 408.71 | |  | |  |
|  |  | | | BD ASP+ | | 3356.74 | 405.92 | | -1.09 | | < BD ASP- |
|  |  | | | SZ ASP- | | 3719.59 | 408.93 | |  | |  |
|  |  | | | SZ ASP+ | | 3652.97 | 406.80 | | -0.16 | | < SZ ASP- |
| Right Caudate * | F (1, 521) = 13.73, *p* = .000 | | | HC ASP- | | 3994.77 | 433.33 | |  | |  |
|  |  | | | HC ASP+ | | 3988.66 | 425.31 | | -0.01 | | < HC ASP- |
|  |  | | | BD ASP- | | 4047.92 | 428.40 | |  | |  |
|  |  | | | BD ASP+ | | 3642.44 | 425.48 | | -0.96 | | < BD ASP- |
|  |  | | | SZ ASP- | | 4022.76 | 428.62 | |  | |  |
|  |  | | | SZ ASP+ | | 3830.12 | 426.40 | | -0.45 | | < SZ ASP- |
| Right Putamen | F (1, 521) = 5.64, *p* = .018 | | | HC ASP- | | 5818.67 | 533.24 | |  | |  |
|  |  | | | HC ASP+ | | 5656.92 | 523.38 | | -0.31 | | < HC ASP- |
|  |  | | | BD ASP- | | 5793.28 | 527.18 | |  | |  |
|  |  | | | BD ASP+ | | 5648.15 | 523.59 | | -0.28 | | < BD ASP- |
|  |  | | | SZ ASP- | | 5902.14 | 527.46 | |  | |  |
|  |  | | | SZ ASP+ | | 5732.60 | 524.71 | | -0.32 | | < SZ ASP- |

Subgroup of participants who have experienced asphyxial-birth complications (ASP+); subgroup of participants who have not experienced asphyxial-birth complications (ASP-); HC (healthy controls); BD (bipolar disorder spectrum); SZ (schizophrenia spectrum)

^a^ Adjusted for age and sex; All *p* values significant at a false discovery rate of 5%

^b^ *d* = Cohen’s *d* effect sizes for ASP+ within-group

^c^ < ASP- implies significant reductions relative to not having experienced asphyxia-related OCs.

* Significant ASP by group interaction for the left and right caudate volumes.
